# River Management for Local Governments in China: From Public to Private

**DOI:** 10.3390/ijerph15102174

**Published:** 2018-10-04

**Authors:** Jiangfan Liu, Xiongzhi Xue

**Affiliations:** 1 College of the Environment and Ecology, Xiamen University, Xiamen 361102, China; jiangfan.liu@hotmail.com; 2 Coastal and Ocean Management Institute (COMI), Xiamen University, Xiamen 361102, China

**Keywords:** PPP, integrated river management, non-standardization, operation framework

## Abstract

The Public and Private Partnership (PPP) model has been used to provide public services and goods. In China, local governments are willing to use the PPP model in many public services, such as integrated river management (IRM) projects, due to ease fiscal budget and the improved access to technology from the private sector. However, there has not been any specific discussion in the literature for applying the PPP model to IRM projects. In this study, we find that the PPP model results in the non-standardization of IRM projects. Our research paper builds the PPP operation framework for IRM projects. Our findings suggest that while the environmental quality evaluation system created in contracts for government payment seems to be optimal for protecting the public interest, it actually strains the partnership between the two parties and so its implementation should be considered on a case by case basis. Since the history of IRM projects using the PPP model is short, the actual performances of these types of projects has not yet been demonstrated. Local governments should be cautious about adopting the PPP model for such projects, and private companies should be cautious about their involvement. Our research will garner more scholarly attention to the application of the PPP model in complex projects.

## 1. Introduction

In China, river pollution is an area of public concern. The rapid development of urbanization and the inefficiency of treatment facilities in rural areas have led to the influx of large amounts of pollutants into rivers. These pollutants include not only COD, ammonia, and nitrogen, but also heavy metals, which seriously affect the water quality and food safety [1,2,3]. The Chinese government has implemented integrated measures to control pollution [4], such as reducing the pollution caused by adjusting the industrial structure, constructing sewage treatment facilities, controlling non-point source pollution, and other comprehensive control measures [5,6]. In these measures, Public and Private Partnerships (PPP), which are mostly Build-Operate-Transfer (BOT), are used in sewage treatment [7] through local governments granting concession contracts to the private sector [8]. It is difficult to apply the PPP model to other types of pollution control projects due to the lack of a suitable financial mechanism.

In order to standardize PPP projects and encourage local governments to carry out PPP projects, the National Development and Reform Commission (NDRC) and Ministry of Finance of the People’s Republic of China (MOF) have established the PPP information platform and statistical system. The MOF, the government budget expenditure management department, offers a comprehensive statistical scale of PPP projects. The PPP projects database can be found in China’s PPP Integrated Information Platform Project Database, which is maintained by China’s PPP Center and was established by the MOF. The 8th Report from China’s PPP center shows that there were 14,424 projects in total, with an accumulated investment of 18.2 trillion Yuan (RMB) [9].

After nearly two years of practice, and with the support of the Chinese central government establishing policies used to promote the PPP model, more local governments preferred to use the PPP model for comprehensive water environment management projects, as well as the management of river and urban black smelly water management [10]. Some of the projects introduced environmental quality performance assessments into the government payment terms of the PPP agreement, which meant that the government would not pay if the environmental quality did meet the specified objectives. Although the PPP model has obvious advantages in such projects, because of its complex operating mechanism, risk allocation, and uncertain issues, the local governments encountered many problems with it.

There is much criticism of the PPP model, including the facts that it can be used to hide government debt and promote unreasonable risk allocation, lacks sustainable supervision, and results in inaccurate demand forecasts [11,12,13], leading to the failure of the PPP projects. So far, there has not been any specific discussion in the literature on the use of the PPP model for IRM projects; this lack includes a discussion on integrated projects, and not only for the application of the PPP model to a single infrastructure project. Therefore, it is necessary to learn how the integrated project uses the PPP model in practice. In order to find evidence, we collected cases from 2014 to 2017, built an optimal operation framework of the PPP model for IRM projects, and discusses the outcomes with current literatures. Local governments attempting to apply the PPP model can benefit from this study.

## 2. PPP Model Review and the Current Situation of the PPP Model in China

The rise of the PPP model can be attributed to its use as a financing tool; it allows for the introduction of social capital investments to ease the pressure on a large number of infrastructure investment expenditures, solving government budget deficits. Another reason for the PPP model’s use is that the public obtains private technology and operational management capabilities to improve the quality and efficiency of public services [14,15].

Previous studies have generated many definitions of the PPP model, and even some debate, but there is no unified PPP model definition because of the various political, economic, legal, cultural, and industrial backgrounds found around the world [16]. The PPP model is a contractual agreement or arrangement between public and private sector to provide public goods and services ([17,18]. The U.S. National Council for Public-Private Partnerships says that “A PPP is a contractual arrangement between a public agency (federal, state or local) and a private sector entity. Through this agreement, the skills and assets of each sector (public and private) are shared in delivering a service or facility for the use of the general public. In addition to the sharing of resources, each party shares in the risks and rewards potential in the delivery of the service and/or facility.”

The openness of the public sector results in the number of PPP application areas and whether PPP is suitable in certain public sectors. The threshold implemented by the government for the private sector should be considered. Based on the incomplete contract and property rights theory, Hart [19] concluded that the PPP model should be applied for public services, where it is easier to write contracts for service provisions. Bentz et al. [20] emphasized the use of asymmetric information to show when governments should buy assets or services from private businesses. There is much research on the PPP model used in the construction industry, which has many soft requirements generated by the government that can be provided through the private sector, such as management system improvements. Tang et al. [14] collected the previous studies related to the PPP model and classified these PPP studies. Risk allocation or risk transfer between public and private entities plays an important role [21,22,23,24], and many researchers stated that risk allocation affects the value of the PPP project assessment. Some studies focused on critical success factors in PPPs [25].

In the water sector, privatization or the PPP model has been widely used in many countries, so there are many studies that examine the link between ownership and performance. Tong et al. [8] gave a comprehensive analysis of urban sewage treatment in China and concluded that the definition of the relationship between the government and market could affect the performance of the PPP project. Renzetti et al. [26] found no compelling evidence of private utilities outperforming public utilities; Walter [27] found other studies linking ownership and performance from France, the U.K., and U.S. Berg and Marques [28] reviewed 47 studies focusing on ownership and found that there were no obvious relationships between ownership and performance. For water utilities in Spain, Suarez-Varela et al. [29] showed that private management was more efficient in the use of labor input, but less efficient at managing operational costs.

Even though enough studies could guide the government and private sector in their execution of certain PPP projects, some barriers exist to prevent local governments from successfully applying the PPP model [30,31], such as a lack of complex project management capabilities. IRM projects include complexities, such as the engineering complexity, which encompasses many different kinds of projects, various types of technical support, and the need for multi-sector cooperation [32], which means that the responsibilities for river management are distributed to different local public sectors, such as the river sector that manages the water resources or the environmental protection sector that manages the water quality.

There is currently no detailed analysis on the conditions and implications of the PPP model in IRM projects. We used existing cases and created a comprehensive analysis using the key contents of the PPP model.

**Figure 1:**
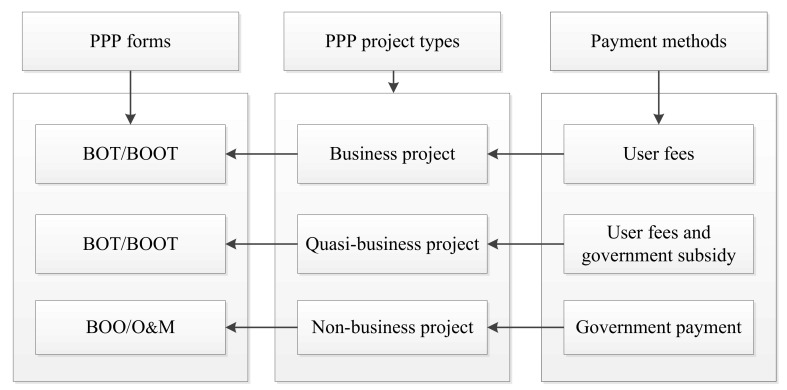
The PPP forms from the NDRC (2014) in China.

In China, the first PPP project was perhaps the Laibin B power station, where a foreign firm from the private sector took part in the project [33]. In order to regulate and stimulate the private sector to enter into the public sector, the NDRC and MOF acted as the supervisors and stated that PPP model could be applied to public facilities, which includes transportation, environmental protection, gas, water, power, heat, waste treatment, hospital, and education. The NDRC also suggested a guideline for PPP forms in order to give advice from the public sector in choosing the proper forms to submit with an application (Figure 1), such as the Build–Operate–Transfer (BOT), Build-Own-Operate-Transfer (BOOT), Transfer-Operate-Transfer (TOT), and Operations & Maintenance (O&M) forms. There are other popular PPP forms around the world, such as the private finance initiative (PFI) in the U.K., the concession form in France, and the private participation in infrastructure (PPI) form of the World Bank. Figure 1 shows that public sector chooses a PPP form for a specific project depending on the payment method.

## 3. Application of the PPP Model to IRM Projects

China’s PPP center classifies IRM projects under eco-construction and environmental protection. In order to facilitate the research, it was necessary to define the PPP model for an IRM project. The concept of IRM is not new, as it is the most important management concept and engineering measure for dealing with water pollution in recent years. Integrated measures are mainly implemented in the upper, middle, and lower reaches of rivers. Engineering measures control polluting from the source to the end of the river and mainly include pollution interception, sewage treatment, river dredging and remediation, wetland construction, ecological restoration, and other types of projects.

Therefore, based on the Project Database, we chose “water environmental management, river basin management, and river ecological restoration” as the keywords to select projects. We set the criteria for the case selection because some projects do not really belong to environmental management; they are only clean-up actions that can temporarily improve the quality of the river and do not accomplish environmental restoration.

For quantitative and qualitative analysis, we collected contracts and the tender notices of the studied projects and then used content analysis to extract information from these records. The content analysis focused on public and private concerns. Based on the content analysis results, we then developed our discussion and conclusions from the perspectives of the local government and private sector.

Nearly 50 projects were collected, but some projects were removed because of unmatched objects or little information. Since PPP projects related to environmental governance have been able to receive government budget approval, some land development projects were packaged with environmental projects and utilized the PPP model.

We collected information from 18 projects from 2014 to 2017, and there was no specificity in terms of the area and scale. The first case of an IRM project using the PPP model appeared in 2014 in a project for the Nanming River. The first case based on the environmental quality performance evaluation method involved the NaKao River. Before the Nanming River project, the government implemented projects that were contracted out one by one, rather than bundling all the projects that affected the river quality improvement to one company in the private sector. As Grimsey et al. [18] suggested, the essence of a PPP is that the public sector does not buy an asset, it is instead purchasing a stream of services under specified terms and conditions.

### 3.1. Payment Methods

Table 1 shows that the payment methods include user fees, user fees and government subsidies, and government payments. Traditionally, local governments adopted “user fees” to cover the costs of projects. For the IRM PPP model, however, the sewage treatment plant, as one of the measures, is packaged together with other projects that are government payment-funded projects. The government payment method is the most commonly adopted approach (Table 2).

### 3.2. Contract Period

There are few cases of a contract duration exceeding 20 years, even from the removed cases; the minimum contract period for samples in this study was 10 years. Local governments consider the contract duration mainly in terms of the annual amount of the local government budget that can be arranged and the payback period for the private sector investment. The long-term contractual period actually appears to be monopolized for a certain period of time, which increases the government’s risk and undermines the public interest.

### 3.3. Tender Option

The project implementation agency is regulated by the Tendering Law and should give priority to open tendering to obtain bidders for public services. In practice, local governments adopt open tendering and competitive negotiation to choose private sector businesses, that the agency uses open tendering as the main way (Table 2). There are preconditions for choosing competitive negotiations, such as the complexity of the project and finding less eligible companies in the private sector.

## 4. Basic PPP Model Operation Framework of IRM Projects

Since local governments do not have a legal representative to directly invest the SPV as a stakeholder, a local government-owned company serves as the representative of the public sector. The regulation implemented by the government allows state-owned company as private sector for PPP projects. Generally, public and private entities set up the SPV using equity cooperation. The private sector has the responsibility for the project financing.

After the bidding process is organized by the government, the private sector business that wins the bid should make a contract with the government. Generally, the PPP project contract consists of two parts: a cooperation agreement signed by the government and private sector and a franchise or service agreement signed by the government and SPV. Both documents have legal validity. Then, the SPV obtains the franchise rights to finance, design, construct, and operate the project, and transfers control of the project to the local government at the end of the contract period. During the bidding process, negotiation over the special terms of the contract is complicated because it determines how the risk is distributed between the parties. Besides the risk allocation, the PPP model of an IRM project should specify how evaluate the service provided by the private sector and how pay according to the assessment results.

**Figure 2:**
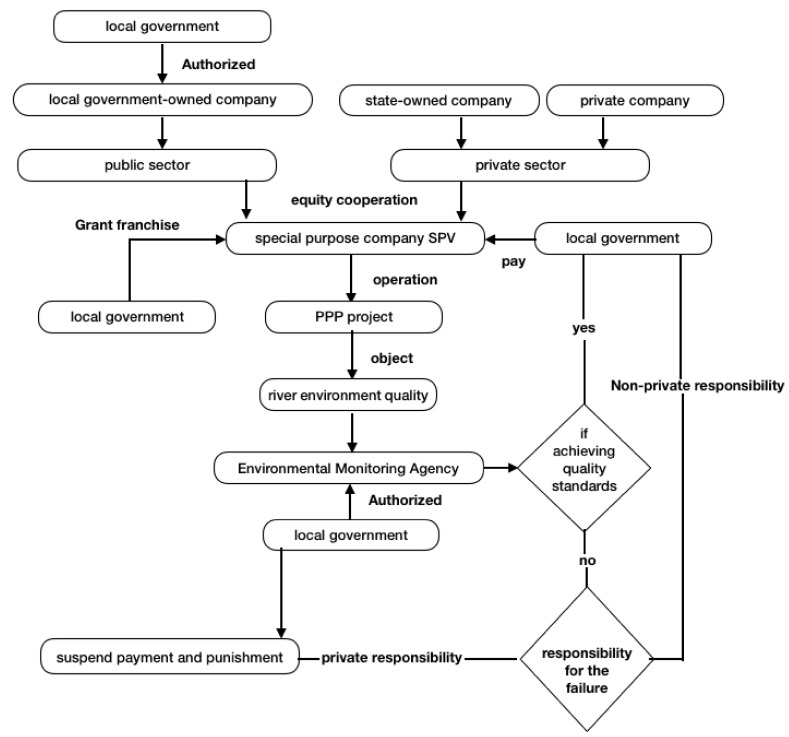
PPP operational framework.

Under the principle of fairness, local governments generally authorize third-party agencies and environmental monitoring agencies to evaluate the operational effectiveness of PPP projects. If the evaluation results achieve the contractual requirements, the local governments comply with the contract to pay; if the project objectives cannot be achieved, a third party analyzes the reasons and then makes a judgement of which party is responsible (Figure 2).

## 5. Findings

### 5.1. The Applicabilty of the PPP Model to IRM Projects

There is no doubt that IRM belongs to the public service. Since specific levels of environmental quality are quantifiable, it is reasonable for private parties to provide environmental improvement services under the PPP model. Bundling is the basic characteristic of the PPP model. The PPP model for IRM projects includes bundling the building and operational stages, as well as bundling different projects together, such as sewage treatment, pollution interception, landscaping, or pure management investment. There is an indirect relationship between the asset operation output and public good, i.e., sewage treatment assets treat polluted water to meet standards, but that does not mean that the river environment can meet the quality standard since there are many issues that affect the environment.

Due to the bundling of multiple projects together, the cases we examined had larger investments, which made their outcomes more uncertain than those of general PPP projects. The governance measures for each river vary due to its economic development, pollution sources, and functional zoning. In addition, the implementation of the PPP model requires the contributions of the local budget, project management, and other factors, making it difficult for local governments to carry out IRM PPP projects because they test the government’s comprehensive management capabilities. Before introducing the PPP model for IRM projects, local governments should research the fiscal budgeting, risk control, performance appraisal, and private returns. Engaging a professional PPP consulting company to assist local governments is necessary. Local governments should also study long term project management issues, and attempt to predict possible challenges and take measures to address them [34]. They should also evaluate the impact on the local government’s long term budget.

The survey also shows that not all local governments have adopted the PPP model in carrying out IRM projects. Non-standardization, over-complexity, and the excessive capabilities of local government’s project management may be factors that hinder the promotion of the PPP model. Therefore, when considering the adoption of the PPP model, local governments should be cautious and examine the actual context of the project.

### 5.2. The Option to Select the Proper Private Sector

Since IRM projects include various types of technologies, they require higher level skills from the private sector. When selecting the appropriate private sector, local governments require that private sector should have a history of operating cases, engineering qualifications, and financing capabilities. It is difficult for a single company to meet all of the requirements. There are 2 solutions to these issues: businesses can engage in a consortium bid, where multiple private firms with different functions present joint bids, or firms can merge into a kind of comprehensive environmental management service provider, with financing, technical services, construction, and operations for the project. Another way is that through equity acquisition, a form of group company owning various companies with different functions during the whole life of a project, and thus enhancing competitiveness.

As mentioned before, state-owned companies also belong to the private sector in China. State-owned companies have relatively lower financing costs and undertake more risks, making them more competitive than purely private companies. Local governments that choose state-owned companies could reduce their political risks. The proportion of state-owned companies in the private sector that have bid on PPP projects has gradually increased, not only in the area of IRM. Many discussions about this issue have mentioned that the risk of local government investment has not been substantially transferred, and it will undermine the development of the private economy.

### 5.3. Risk Allocation between 2 Parties

Risk allocation is the core of PPP projects. However, no matter how risks are transferred from government to private entities, the government still undertakes the ultimate responsibility for a project’s operational risk [35]. From the perspective of the private sector firms, bidding on IRM PPP projects mean that they do not need to go through negotiations with the government. However, the corresponding risks also greatly increase. In particular, when local governments adopt a contract payment method with environmental quality in the evaluation system, it transfers some of the environmental quality risks to the private company, and the uncertainties affecting the environmental quality can be numerous, since there are many uncontrollable factors, such as the weather. Before participating in IRM PPP projects, private sector firms should conduct self-assessments on their technological capabilities and risk-undertaking ability. Private firms should undertake financial risks. If it is impossible to define most risks and quantify those risks, private companies should be cautiously involved in the IRM PPP project.

Therefore, contracts alone, which are often imperfect and incomplete, are not enough, and local governments should continuously supervise the private sector [36]. Some of the cases examined in this research showed that the private sector had complete ownership of the SPV. The UK’s latest PPP model (PF2) emphasizes that the government should act as a minor stakeholder in the SPV, which can increase the government supervision of the project and project transparency [37]. Since a project is usually entirely financed by the private sector, if a local government invests in it as a major shareholder, the project would not meet the fundamental principle of PPP risk allocation. It is necessary for the government to become a minority shareholder. Continuous supervision is necessary due to the integrated management of rivers, which involves multiple administrative departments, such as environmental protection, water conservancy, agriculture, and forestry; the implementation of a project requires the approval of different departments. The participation of the local government can reduce the difficulty of negotiations.

### 5.4. The Payment Method Options

The cases examined in this research suggest that local governments have already applied various payment methods based on performance evaluations. After introducing the performance appraisal system for IRM PPP projects, local governments should pay more attention to the improvement of the river’s environmental quality, rather than the quality of the individual project. However, some local governments still adopt traditional payment methods. In these cases, local governments have adopted two main payment methods in practice: (1) single service fees that are totally based on the Performance Evaluation (P1) and a (2) 2-phase payment method consisting of usability service fees and operational and maintenance performance service fees (P2), where the government pays for the availability asset and then pays for the service when the river quality meets the standard.

However, P2 has attracted the attention of some experts and policy-makers, who argue that usability service fees lead to the overinvestment at the building stage and that governments should use the single service fees. Since P2 is an innovative approach that only emerged in the past 3 years, the previous papers have not discussed the argument from the perspective of microeconomics. Under the incomplete contract method [19], at the end of building stage, the usability service fee has more incentive to increase investment than P1. Improving the quality of the asset would decrease the cost of operation. The reason for this is that private investments in improving the quality of assets are paid by the government, so the private sector does not consider the cost of project’s improvement and renegotiates with the government for the contract adjustment. However, the premise is that the government could observe the information of the project, such as the cost and the quality. Generally, the third-party appraisal agencies are jointly agreed upon by the government and private partner to identify the investment and asset quality at the end of the building stage.

Our research shows that the payment method, choice of the private sector firms and ownership structure of the SPV, and differences among local governments make the PPP Model non-standardized.

### 5.5. Interest of the Local People

Local people are the taxpayers, and also the terminal PPP project payers, whose welfare should be considered by the government when it implements the PPP model. For IRW projects, the sewage treatment price can directly reflect the social cost, while other fees from the parts of IRM project packages are harder to collect from local people because they are difficult to calculate. Using the open tender system, the government can choose the lowest cost bidder to achieve value for its money. Online information has shown that it is hard to find obvious evidence that the PPP model would increase or decrease the cost of IRM projects. The disadvantage of a price war is that winner has the possibility to cut the building or operating costs, which then affects the quality of the project and the operational outcome.

However, there are two signals that should not be ignored. In order to win the bid, private sector firms often use price wars to maliciously compete with prices below the investment cost to obtain the project franchise. They then take advantage of their monopoly of the project to force the government to raise prices or give other support, which is available to the firms through the price increase clause in the contract. Another signal is that the market supervisor issues a policy to allow the local government to dynamically adjust water treatment fees. Since the application of the PPP model IRM projects has a short history, it is hard to estimate the cost-effectiveness of the PPP model using the available cases. There are no academic papers that discuss this issue.

## 6. Conclusions

This research provides the evidence that IRM projects can be used with the PPP model. With the background of the PPP boom, local governments that implement the PPP model in various public service areas can solve the problems of excessive local government debt and increase the efficiency of project operations, as well as transform government management methods.

This paper summarizes a PPP operational framework in applicability, which could provide a reference for local governments that want to implement IRM projects using the PPP model or private sector that want to participate in PPP projects. Although the basic operational framework of the PPP model is mature and commonly practiced, it is difficult to find information about it in IRMS projects because of the non-standardized nature of the project. Therefore, before choosing the PPP model for IRM projects, local governments should not excessively consider the advantages of the PPP model and understand that the PPP model makes projects more complex. Both parties have to seriously consider whether the partnership building in PPP models is proper, rather than focusing on the short-term benefits.

The adoption of an environmental quality performance appraisal system is optimal, but it increases the difficulty of risk allocation between 2 parties. If both parties do not have sufficient experience, this method should be used with caution because the government still has the ultimate responsibility for environmental quality. The local government should supervise the whole lifecycle of a PPP project, and as SPV’s minor shareholder can yet be a way to perform duties.

Since this new type of PPP model is still being explored, the research in this paper was superficial, and there were deficiencies in the data and analysis. This research can, however, provide a reference for local governments and private sector to cooperate via the PPP model, and the complex PPP model could attract more scholars’ attention and result in further research.

## Figures and Tables

**Table 1:** Explanation of the payment methods, tender options, and Special Purpose Vehicle (SPV) equity structure.

Payment method	Government payment	P1: Based on Performance Evaluations
		P2: Consists of a usability service fee and operational and maintenance performance service fees
		P3: Traditional government payment
	P4: User fees and government subsidies	If user fees did not cover the cost and returns for the private sector, the government grants the subsidy for private financing
	P5: User fees	Revenue from the users who access to the services
Tender option	T1: Open tendering	Open to all qualified bidders
	T2: Competitive negotiation	Negotiating the pricing and terms surrounding a particular transaction
SPV equity structure	Public sector: private sector	

**Table 2:** The information of cases in this study.

Case	River Name	Investment Unit: ×10^8^ RMB	Contract Period (Construction Period), Unit: Years	Payment Method (P(n))	Tender Option	SPV Equity Structure
1	Jimo Moshui River	17.29	10 (1.5)	P3	T1	0:100
2	Yuhuan Yukan River	6.57	30	P3		0:100
3	Huaian Baima River	10.00	10 (2)	P2		10:90
4	Xiuwu Yunliang River	>6.00	10 (2)	P2		-
5	Lishui Liyang River	7.50	15	P3		5:95
6	Nanzhao Huangya River	34.97	13 (2)	P4		20:80
7	Jiaocheng Ciyao River	8.55	15 (3)	P3		10:90
8	Beijing Xinfeng River	41.48	20	P2		1:99
9	Dushan Jiushijiutan River	45.65	24	P1		11:89
10	Guiyangshi Maijia River	17.90	15	P2		0:100
11	Anshan Center River	34.80	18	P2		5:95
12	Quanzhou Batou River	6.84	20 (3)	P2		20:80
13	Nanning Nakao River	10.01	10 (2)	P1	T2	10:90
14	Jingmen Zhipi River	31.24	30	P3		10:90
15	Nanning Shajiang River	24.61	15 (2)	P1		20:80
16	Ruyang Beiru River	8.77	10 (2)	P2		0:100
17	Tai’an Wen River	12.80	12 (2)	P3		5:95
18	Cangzhou Qingxi River		12 (2)	P2		20:80
